# P-1069. Comparative Antibacterial Activity of Hypochlorous and Polygalacturonic + Caprylic Acids in an *In Vitro* Wound Biofilm Model

**DOI:** 10.1093/ofid/ofae631.1257

**Published:** 2025-01-29

**Authors:** Bahgat Z Gerges, Issam I Raad, Ying Jiang, Joel Rosenblatt, Y Lan Truong

**Affiliations:** MD Anderson UT, Missouri City, Texas; MD Anderson UT, Missouri City, Texas; MD Anderson, UT, Houston, Texas; MD Anderson UT, Missouri City, Texas; UT MD Anderson Cancer Center, Houston, Texas

## Abstract

**Background:**

Therapeutic agents which impart minimal inflammatory responses are needed to disinfect microbially contaminated chronic wounds. Polygalacturonic + caprylic (PG+CAP) and hypochlorous (HOCl) acids have been shown to be well tolerated potent antimicrobial agents for eradicating a broad range of microbial biofilms in standard *in vitro* biofilm models. However, standard microbiologic biofilm eradication models do not present the complex natural environments of chronic wound beds which contain significant quantities of human proteins. In this study we compared the efficacy of PG+CAP and HOCl against resistant wound biofilm bacterial pathogens in a 3-dimensional fibrin gel wound biofilm model, which is more realistically simulates a colonized chronic wound.

**Figure:**
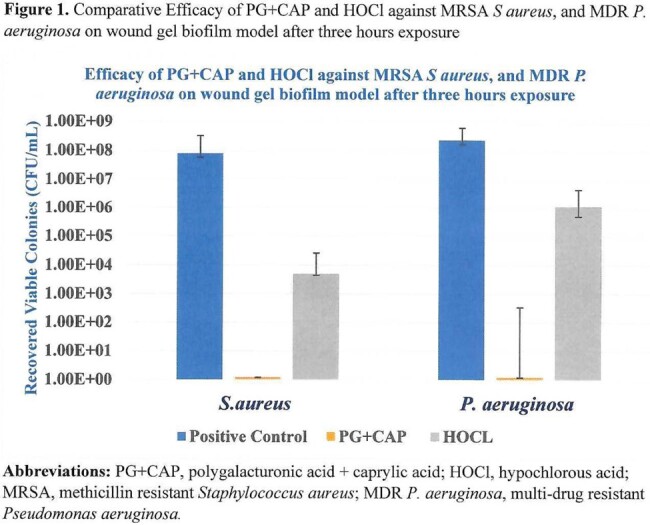

**Methods:**

A fibrin gel biofilm eradication model was used to quantify antibacterial efficacy. Forty-eight hours mature biofilms of *Staphylococcus aureus* and *Pseudomonas aeruginosa* were allowed to form on fibrin gels creating wound biofilm models. They were then exposed to 1% PG+ 0.8% CAP or 400 ppm HOCl for three hours. Following exposure, the wound gels were sonicated in neutralizer broth, then serially diluted, plated and counted to enumerate viable colonies (CFU/mL). Positive controls were not exposed to antimicrobial agents. Statistical comparisons of six-replicates for each group were performed using Wilcoxon rank sum test (p<0.05 is considered statistically significant).

**Results:**

**Figure 1** presents the median number of CFU/mL recovered from controls and wound biofilms exposed to PG+CAP, and HOCl.

**Conclusion:**

PG+CAP completely eradicated *S aureus* biofilms with greater than 7-log reduction in CFU/mL versus positive control, while HOCl produced a 4.2 log reduction. The CFU/mL difference between PG+CAP and HOCl was significant (*P*= 0.0028). For *P. aeruginosa*, PG+CAP and HOCl produced 8.28 and 2.32 log reductions versus positive control respectively with a significant difference in CFU/mL between them (*P*= 0.0043). These results suggest microbiologic testing in wound biofilm models may be important to realistically test the efficacy of antimicrobial agents for chronic wound care. These results suggest PG+CAP merits further testing for disinfecting contaminated chronic wounds.

**Disclosures:**

**All Authors**: No reported disclosures

